# ATP Secretion and Metabolism in Regulating Pancreatic Beta Cell Functions and Hepatic Glycolipid Metabolism

**DOI:** 10.3389/fphys.2022.918042

**Published:** 2022-06-21

**Authors:** Jing Li, Han Yan, Rui Xiang, Weili Yang, Jingjing Ye, Ruili Yin, Jichun Yang, Yujing Chi

**Affiliations:** ^1^ Department of Endocrinology, Beijing Chao-Yang Hospital, Capital Medical University, Beijing, China; ^2^ Key Laboratory of Cardiovascular Science of the Ministry of Education, Center for Non-coding RNA Medicine, Department of Physiology and Pathophysiology, School of Basic Medical Sciences, Peking University Health Science Center, Beijing, China; ^3^ Beijing Key Laboratory of Diabetes Research and Care, Beijing Tongren Hospital, Capital Medical University, Beijing, China; ^4^ Department of Central Laboratory and Institute of Clinical Molecular Biology, Peking University People’s Hospital, Beijing, China; ^5^ Key Laboratory of Trauma and Neural Regeneration (Peking University), National Center for Trauma Medicine, Trauma Medicine Center, Peking University People’s Hospital, Beijing, China; ^6^ Beijing Key Laboratory of Diabetes Prevention and Research, Center for Endocrine Metabolic and Immune Disease, Beijing Luhe Hospital, Capital Medical University, Beijing, China

**Keywords:** ATP, purinergic P2 receptor, insulin secretion, glucolipid metabolism, mitochondrial dysfunction

## Abstract

Diabetes (DM), especially type 2 diabetes (T2DM) has become one of the major diseases severely threatening public health worldwide. Islet beta cell dysfunctions and peripheral insulin resistance including liver and muscle metabolic disorder play decisive roles in the pathogenesis of T2DM. Particularly, increased hepatic gluconeogenesis due to insulin deficiency or resistance is the central event in the development of fasting hyperglycemia. To maintain or restore the functions of islet beta cells and suppress hepatic gluconeogenesis is crucial for delaying or even stopping the progression of T2DM and diabetic complications. As the key energy outcome of mitochondrial oxidative phosphorylation, adenosine triphosphate (ATP) plays vital roles in the process of almost all the biological activities including metabolic regulation. Cellular adenosine triphosphate participates intracellular energy transfer in all forms of life. Recently, it had also been revealed that ATP can be released by islet beta cells and hepatocytes, and the released ATP and its degraded products including ADP, AMP and adenosine act as important signaling molecules to regulate islet beta cell functions and hepatic glycolipid metabolism via the activation of P2 receptors (ATP receptors). In this review, the latest findings regarding the roles and mechanisms of intracellular and extracellular ATP in regulating islet functions and hepatic glycolipid metabolism would be briefly summarized and discussed.

## 1 Introduction

DM is a metabolic disease characterized by chronically elevated levels of blood glucose. Among the diabetic patients, type 1 diabetes mellitus (T1DM) accounts for about 5%–10% while T2DM accounts for more than 90%, and other types of diabetes including gestational diabetes (GD) and Maturity Onset Diabetes of the Young (MODY) account for only a small proportion (Gonzalez-Franquesa and Patti, 2017). According to the International Diabetes Federation (IDF) diabetes map, it was estimated that there were 415 million diabetic patients in the world in 2015, and the number was expected to reach 642 million by 2040 (Cho et al., 2018). It had been reported that in China, the prevalence of diabetes among adults was 12.8%, and the prevalence of pre-diabetes among the adult population was 35.2% in 2017 (Li et al., 2020). The recent study estimated the prevalence of diabetes and prediabetes in China were 12.4% and 38.1% separately in 2018 (Wang L. et al., 2021).

So far, it had been widely acknowledged that islet β cell dysfunctions and insulin resistance are the two key factors causing T2DM (Bensellam et al., 2018; Holst et al., 2018; Zhang et al., 2019). Generally, when insulin resistance occurs, islet β cell compensates for insulin insufficiency by increasing insulin secretion ability of individual cell or by proliferating, once pancreatic islet β cells fail to compensate for insulin resistance, hyperglycemia will be established (Biddinger and Kahn, 2006; Bensellam et al., 2018; Holst et al., 2018). Clearly, how to protect or even restore the normal functions of islet β cells is critical for delaying or even curing diabetes.

Disorders of glucose and lipids metabolism caused by genetic and/or environmental factors triggers diabetes. Increased hepatic gluconeogenesis is a common decisive event in the pathogenesis of T2DM (Biddinger and Kahn, 2006). In this process, abnormal increase in hepatic glucose production, mostly derived from glycogenolysis and gluconeogenesis, plays the decisive role in the development of fasting hyperglycemia. The elevation of gluconeogenic gene expressions in the liver resulting from insulin secretion deficiency and/or insulin resistance leads to the abnormal increase in hepatic gluconeogenesis, which finally increases fasting blood glucose. In human, increased hepatic gluconeogenesis but not overall glucose production always precedes the onset of T2DM (Jin et al., 2013; Yang et al., 2019).

ATP plays important roles in regulating a number of biological processes, including biosynthesis, signal transporting, genetic information processing, cell mobility etc. (Chen and Zhang, 2021; Johnson et al., 2019). Impairment of ATP production might cause many diseases including cardiovascular diseases, metabolic diseases and the occurrence of aging (Qi et al., 2018; Zheng et al., 2018). In this review, the roles and mechanisms of ATP in the pathogenesis of the metabolic disorders would be briefly discussed, with a focus on islet β cell functions and hepatic glycolipid metabolism.

## 2 Reduced ATP Production and Dysfunction of ATP Signaling Associates With Diabetes

### 2.1 ATP Synthesis

ATP is mainly synthesized in mitochondria by ATP synthase complex (ATPS). ATPS consists of two parts, F0 region with proton-transporting function while F1 region in the inner membrane with ATP synthesis function. F0 region is mainly composed of three subunits: b, c, and d, and F1 part is mainly composed of five subunits of α, β, γ, δ, and ε in mammalians (Sabbert et al., 1996; Hahn et al., 2018). ATPS utilizes the proton gradient (mitochondrial potential) formed by oxidative phosphorylation (OXPHOS) across the intermembrane space and matrix of mitochondria to synthesize ATP (Sabbert et al., 1996; Hahn et al., 2018). In animal models such as streptozotocin (STZ)-induced type 1 diabetic, and obese type 2 diabetic mouse livers, the expression of beta subunit of ATPS (ATPSβ), the key catalytic subunit of ATPS, is decreased with reduced ATP content, hinting that decreased ATPSβ expression or activity may play key roles in the development of hepatic glycolipid metabolism (Vendemiale et al., 2001; Wang et al., 2014a).

Although ATP was canonically produced by ATPS in mitochondria, recent study found that the plasma membrane also exists the ecto-F0F1-ATP synthase (ectopic ATP synthase) in multiple cells (Moser et al., 2001; Mangiullo et al., 2008; Fu and Zhu, 2010; Wang et al., 2019). In rat hepatocytes, ectopic ATP synthase is encoded both by nuclear and mitochondrial gene, which might be assembled in mitochondria then transported to plasma membrane, and regulate HDL uptake on cell surface (Rai et al., 2013; Song et al., 2014). Another study in cholestasis also indicated the role of ectopic ATP synthase in controlling cholesterol transport (Giorgio et al., 2010). These indicated the ectopic ATP synthase might also participate hepatic metabolism.

### 2.2 Metabolism of Extracellular ATP

Released ATP by cells can be degraded by soluble or plasma membrane-bound ecto-nucleotidases. There are mainly four ecto-nucleotidases families, including ecto-nucleoside triphosphate diphosphohydrolases (E-NTPDases), ecto-5′-nucleotidase (E-5′-NT), ectonucleotide pyrophosphatase/phosphodiesterases (E-NPPs), and alkaline phosphatases (APs) (Zimmermann et al., 2012). The E-NTPDases are nucleotide-specific and hydrolyze nucleoside triphosphates or diphosphates to nucleoside monophosphates. E-5′-NT is also nucleotide-specific and degrades AMP to adenosine. E-NPPs hydrolyze nucleoside triphosphates and diphosphates, dinucleoside polyphosphates, and other substrates but not AMP. Beside hydrolyzing triphosphates, diphosphates, monophosphates and pyrophosphate, APs also hydrolyzes amount of monoesters of phosphoric acid (Zimmermann et al., 2012). The phosphohydrolysis of ATP by ectonucleotidases will stimulate purinergic signaling in hepatic disease including ischemia reperfusion, hepatic regeneration, steatohepatitis (NASH) and cancer in animal models (Vaughn et al., 2014). Overexpression of ectonucleotidases protein such as ENTPD1/CD39 and ENTPD2/CD73 on hepatic stromal cells will result in hepatic metabolic homeostatic integration and immune reactions in the liver (Jhandier et al., 2005; Pommey et al., 2013; Shuai et al., 2021), and genetic knock out of ENTPD1/CD39 leading to insulin resistance and worsen hepatic glucose metabolism (Enjyoji et al., 2008).

**FIGURE 1:**
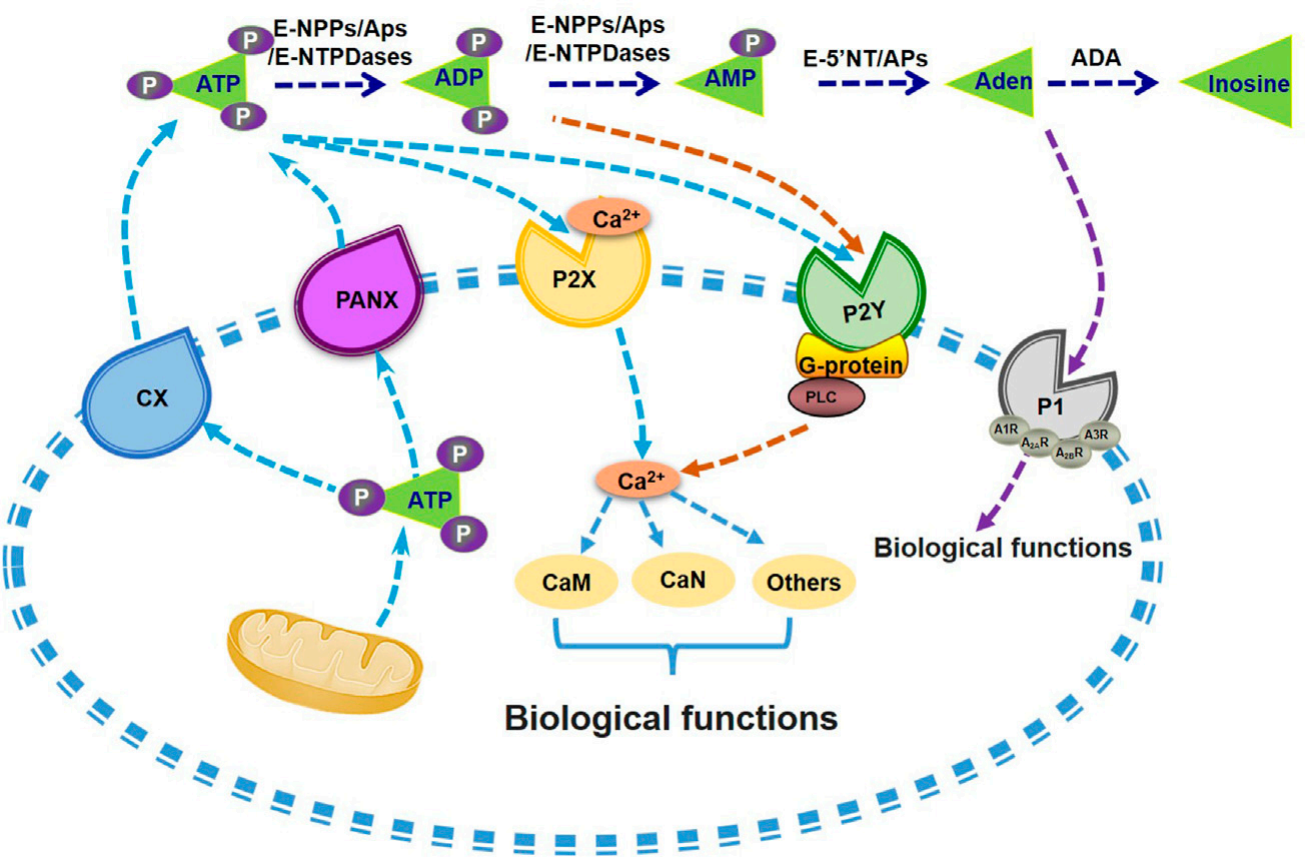
Metabolism of extracellular ATP and its biological functions. ATP is synthesized and then secreted from the cells through CX or PANX channels. Extracellular ATP can be degraded into ADP and AMP by the catalysis of E-NTPDases, APs and E-NPPs. AMP can be further hydrolysed into adenosine by APs or E-5′NT. P1 receptors can be activated by adenosine and promote its influx into the cell. Nucleotides existing in the extracellular also activate P2 receptors, including P2X and P2Y receptors, with the induction of Ca^2+^-CaM signal pathways or G-protein-PLC mediated activation of PI3K, or other biological regulations. Aden, adenosine. APs, alkaline phosphatases. CaM, calmodulin. CaN, Calcineurin. CX, connexin channels. E-5′NT, ecto-5′-nucleotidase. E-NTPDases, ecto-nucleoside triphosphate diphosphohydrolases. E-NPPs, ectonucleotide pyrophosphatase and phosphodiesterases. P1, P1 receptors. P2X, P2X receptors. P2Y, P2Y receptors. PANX, pannexin channels. PI3K, phosphatidylinositol 3-kinase. PLC, phospholipase C.

Adenosine will activate purinergic P1 receptors (P1Rs) including A_1_ (A_1_R), A_2A_ (A_2A_R and A_2B_R), and A_3_ (A_3_R) to participate multiple effects such as stimulating inflammatory cells, generating radical formation, as well as moderating cell metabolic function, and all these P1 receptors belong to the superfamily of G protein-coupled receptors that represent the most widely targeted pharmacological protein class (Trincavelli et al., 2010; Wang S. et al., 2021). In recent years, it had also been reported that P1Rs also play important roles in liver metabolism. Peng et al. (2009) found that ethanol metabolism might induce intracellular and extracellular adenosine accumulation, which triggers ethanol-induced hepatic steatosis through A_1_ and A_2B_ receptors. A_2A_R stimulation would protect from the development of NASH (Alchera et al., 2017), and A_2A_R disruption augment the process of NAFLD in mice (Cai et al., 2018). A_3_R agonist treatment also alleviated NASH in mice (Fishman et al., 2019). Overall, these findings suggest that metabolism of extracellular ATP plays important roles in maintaining glucose and lipid homeostasis (Figure 1). Particularly, the homeostasis of among extracellular ATP, ADP, AMP and adenosine should be a character of metabolic healthy cell.

### 2.3 Extracellular ATP Signaling

ATP was first isolated from muscle and liver extracts by Lohmann in 1929 (Langen and Hucho, 2008), then Geoff Burnstock and colleagues found that ATP is not only a universal intracellular energy carrier but plays also an important role as extracellular signaling molecule (Burnstock et al., 1970; Burnstock, 1972). Extracellular ATP plays crucial biological roles in various tissues. Additional studies showed that ATP as widespread cell-to-cell signaling molecule regulates multiple cell functions (Lazarowski et al., 2011). The mechanisms of ATP release from cells involve in exocytosis, plasma membrane-derived microvesicles, calcium homeostasis modulator 1 (CALHM1), specific ATP-binding cassette (ABC) transporters, volume-regulated anion channels (VRACs), maxi-anion channels (MACs) and membrane channels [connexin (CX), pannexin (PANX)] (Lohman and Isakson, 2014; Dahl, 2015; Vultaggio-Poma et al., 2020).

Extracellular ATP could activate the purinergic P2 receptors (P2Rs) on the cell membrane, including the P2X receptors and the P2Y receptors subfamilies. P2X receptors are cation-selective channels, while P2Y receptors are G-protein-coupled receptors (Cho et al., 2018; Vultaggio-Poma et al., 2020; Volker et al., 2020). The P2X receptor subfamily consists of seven subtypes (P2X1-7), while the P2Y receptor subfamily includes eight members (P2Y1, 2, 4, 6, 11,12, 13, 14), respectively (Alarcón-Vila and Pelegrín, 2019; Vultaggio-Poma et al., 2020). The P2X receptors are mainly activated by ATP, while the typical ligands for P2Y receptors include ATP, ADP and uridine triphosphate (UTP) (Abbracchio et al., 2006; Burnstock, 2007). Among them, the P2Y2 and P2Y11 is dominantly activated by ATP, while P2Y1 receptor is sensitive to ATP and ADP (Abbracchio et al., 2006; Burnstock, 2007). it has been confirmed that extracellular ATP not only acts on P2 receptor to enhance glucose stimulated insulin secretion (GSIS) in rodent and human islets (Richards-Williams et al., 2008; Jacques-Silva et al., 2010), but also regulates glycolipid metabolism in the livers (Wang et al., 2014b).

### 2.4 Distribution of P2 Receptors in Pancreatic Beta Cells and Hepatocytes

It had been reported that several subtypes of P2 receptors including P2X1-4, P2X6-7, P2Y1, P2Y2, P2Y4, P2Y6 and P2Y11-13 are functionally expressed in pancreatic β cells (Petit et al., 2009; Burnstock and Novak, 2013). After glucose challenge, ATP secreted together with insulin in a pulsed manner provides a feedback signal in islet β cells. P2X (but not P2Y receptors) and Ca^2+^ dependent K^+^ channels participate in this potential signal cascade (Bauer et al., 2019). Clarissa et al. found that in pancreatic β cells, ATP could also activate calcium-mobilized P2Y purinergic receptors (Bartley et al., 2019). In general, some P2 receptors facilitate insulin release, while others impair insulin secretion in pancreatic β cells. Previous research conducted in hamster beta cell line (HIT-T15) revealed that long-term activation of ATP-P2X7 signaling pathway inhibited GSIS, and led to cell apoptosis and DNA fraction (Lee et al., 2007; Lee et al., 2008). Moreover, activation of P2X3 and P2Y13 also exerted deleterious effects on insulin secretion in pancreatic β cells (Petit et al., 2009). In contrast, P2Y1 can be activated by Ca^2+^-induced ATP release to depolarize cell membrane and amplify Ca^2+^ signals, augmenting insulin secretion in pancreatic β cells (Hellman et al., 2004; Tengholm, 2014).

In whole rat livers, the transcripts of P2X1-P2X7 receptors were all detectable, while only those of P2X4 and P2X7 were significantly detectable in isolated hepatocytes. In contrast, the other P2X receptors such as P2X5 and P2X6 were hardly detected in isolated hepatocytes (Emmett et al., 2008). Particularly, P2X4 receptor was widely expressed in mouse hepatocytes, rat HTC cells and Huh seven cells. P2X4 was further shown to be localized in the basolateral and canalicular membrane of hepatocytes, where it regulated Na^+^ and Ca^2+^ signals upon ATP stimulus (Emmett et al., 2008). In addition, P2X7 antagonist exhibited beneficial effects on NASH and liver fibrosis (Jain and Jacobson, 2021). In mouse livers, P2Y1, P2Y2, P2Y6 and P2Y12-14 transcripts were significantly expressed, and played different roles in the pathogenesis of fibrosis (Emmett et al., 2008; Ortega et al., 2014). The expression of P2Y2 was positively correlated with hepatocyte proliferation (Prats-Puig et al., 2013), while P2Y6 and P2Y14 had beneficial effects on NAFLD and hepatic inflammation (Jain and Jacobson, 2021). P2Y receptors could also mediate hepatic glycogenolysis, eicosanoid production and regulate cellular Ca^2+^ and cAMP homeostasis (Emmett et al., 2008; Olioso et al., 2019). In hepatocytes, extracellular ATP promoted glycogenolysis (Keppens and De Wulf, 1985), inhibited gluconeogenesis (Asensi et al., 1991) and fatty acid synthesis (Guzman et al., 1996).

### 2.5 Reduced ATP Production is Highly Associated With Diabetes

In islet β cells, chronic exposure to high levels of pro-inflammatory factors (interleukin-1β, tumor necrosis factor-α and interferon-γ), free fatty acids (FFAs) and glucose will inhibit the synthesis of ATP and impair insulin secretion (Poitout et al., 2010; Giacca et al., 2011). Generally, the reduction of ATP synthesis is usually accompanied by an increase in mitochondrial potential, which trigger the excessive production of reactive oxygen species (ROS) and cause oxidative stress to further impair the survival and insulin secretion of islet β cells (Mayr et al., 2010; Wang et al., 2014c). Other studies also revealed that leucine can enhance ATP synthesis in rat islets, human diabetic islets, and islet β cell line (INS-1) by up-regulating ATPSβ, thereby enhancing insulin secretion with or without glucose stimulation (Yang et al., 2004; Yang et al., 2006). Studies have also shown that hepatic ATP synthesis and content were reduced in high fat diet (HFD)-induced type 2 diabetic mice, insulin resistant-patients, and type 2 diabetic patients (Miyamoto et al., 2008; Serviddio et al., 2008; Berglund et al., 2009; Sharma et al., 2009; Szendroedi et al., 2009; Schmid et al., 2011). Moreover, in STZ-induced type 1 diabetic mice and rats with methionine and choline deficient diet (MCD) induced non-alcoholic steatohepatitis, the hepatic ATP contents were also remarkably reduced (Vendemiale et al., 2001; Wang et al., 2014c). These results revealed that the impairment of ATP synthesis is highly associated with pancreatic β cell dysfunctions, and dysregulated hepatic glycolipid metabolism. Meanwhile, the plasma ATP concentration significantly decreased in type 2 diabetic patients compared with control participants detected by intravascular microdialysis technique (Groen et al., 2019). In the diabetic children also observed the reduced blood ATP level (Kniazev Iu et al., 1985).

## 3 ATP Regulates Islet β Cell Functions

In islet β cells, ATP synthesis and release plays key roles in controlling the synthesis and secretion of insulin. Intracellular ATP is the key mediator of GSIS (Seino, 2012). Besides, it also stimulates β cell regeneration and proliferation (Andersson, 2014). Further research revealed that ATP released from β cells could act on purinergic receptors of resident macrophages in islets, which stabilize the islet composition and size to maintain the insulin secretary function of islets (Weitz et al., 2018). Reduction of ATP synthesis is an important feature of mitochondrial dysfunction, and is also a decisive event that causes islet β cell dysfunctions and diabetes (Biddinger and Kahn, 2006; Sha et al., 2020).

### 3.1 Intracellular ATP Stimulates GSIS in Pancreatic β Cell

After meals, circulating glucose is transported to islet β cells by glucose transporter 2 (GLUT2), and is oxidized into pyruvate by glycolysis. Subsequently, the oxidative metabolism of pyruvate in mitochondria increases the intracellular ATP content and the ATP/ADP ratio, which closes ATP-sensitive K^+^ (K_ATP_) channel to depolarize cell membranes and open voltage-dependent Ca^2+^ channel, resulting in the influx of extracellular Ca^2+^. Finally, an increase in cytosolic Ca^2+^ triggers insulin-secretory vesicles to transport and fuse with the cell membrane, eventually releasing insulin particles (De Marchi et al., 2017; Gerencser, 2018). Overall, intracellular ATP is essential for maintaining islet β cell function particular insulin granule exocytosis, and interruption or reduction of mitochondrial ATP synthesis will impair insulin secretion of pancreatic β cells.

#### 3.1.1 Reduction of ATP Synthesis Impairs Pancreatic β Cell Functions

Under obese or insulin resistant condition, chronically increased levels of blood glucose, free fatty acids (FFAs) and inflammatory cytokines will exert deleterious effects on islet β cells, which are called glucolipotoxicity and cytokine toxicity, respectively (Wang et al., 2010; Bellini et al., 2018; Huang et al., 2018; Xue et al., 2018). The toxicities of hyperglycemia, FFAs and inflammatory cytokines in pancreatic β cells are highly associated with inhibition of mitochondrial ATP synthesis. Reduction of mitochondrial ATP production leading β cells dysfunction during in glucose toxicity exposure (Kim et al., 2005a). Long-term exposure to high glucose could cause decreased expression of glucokinase (GCK), reduced cellular ATP production and insulin secretion, and weaken GCK-mitochondria interaction (Kim et al., 2005a). Haythorne et al. (2019) found that hyperglycemia might changing β-cell metabolism, that significantly reduces mitochondrial function and ATP synthesis as well as β cells failure. Reduced ATP synthesis is also involved in the lipotoxicity induced by free fatty acids (FFAs) in β cells. Saturated long-chain FFAs (C20-C22) have deleterious lipotoxic effects on human EndoC-betaH1 beta-cells by inducing hydroxyl radical formation and cardiolipin peroxidation, and causing ATP depletion (Plotz et al., 2019).

Moreover, inhibition of ATP is also involved in cytokine-induced toxicity islet β cells. In islets, IL-1 beta and TNF alpha promoted the generation of nitric oxide which inactivates enzymes such as aconitase and ribonucleotide reductase by formation of iron-nitrosyl complexes. This in turn may reduce the oxidation of glucose and synthesis of ATP (Hahn et al., 2017). In isolated islets of β cell specific Pax6-deficient mice, glucose-induced elevation of cytosolic ATP/ADP ratio is impaired, which leads to blunted insulin expression and secretion (Mitchell et al., 2017). Moreover, in diabetic βV59M mice (a non-obese, eu-lipidaemic diabetes model with suppressed insulin secretion), in which tamoxifen-inducible expression of a constitutively open K_ATP_ channel specifically in pancreatic β cells, the ATP synthesis rate and insulin secretion are markedly reduced in the islet β cells when compared with that in wild type mice (Haythorne et al., 2019).

Mitochondrial ATP synthesis rate and GSIS of islets isolated from Olfactory protein 4 (OLFM4)-deficient mice were significantly enhanced and resistant to HFD-induced-glucose intolerance and insulin resistance. OLFM4 is mainly localized in mitochondria and negatively regulates GSIS by interacting with genes associated with retinoid-interferon mortality (GRIM-19) in Min6 cells (Liu et al., 2018). Kojima et al. (2017) found that inhibition of type 1 taste receptor-3 (T1R3) and calcium-sensing receptor (CaSR) heterodimers, which are the main components of glucose receptors that inhibit the production of ATP and GSIS in islet β cells. The immunosuppressant tacrolimus (rapamycin) is widely used in tissue or islet transplantation. However, an important side effect of long-term use of rapamycin is the inhibition of mitochondrial respiratory function, which reduced mitochondrial ATP production and decreased cellular Ca^2+^ level, thereby blunted GSIS in islet β cells and increased the risk of diabetes (Lombardi et al., 2017). ATPase inhibitory factor 1 (IF1) also negatively regulates GSIS by inhibiting ATP synthesis in islet β cells (Kahancova et al., 2018).

Sirtuins (SIRTs) which have seven isoforms are highly conserved nicotinamide adenine dinucleotide (NAD)^+^-dependent deacetylases and ADP-ribosyl transferases (Han et al., 2019). It has been shown that SIRT4 involved in regulating cellular ATP content and insulin secretion in pancreatic β cells (Haigis et al., 2006). Spaeth et al. (2019) reported that both the pancreas size and islet β-cell functions of mice are controlled by the ATP-dependent Swi/Snf chromatin remodeling coregulatory complex, which interacts with pancreatic and duodenal homeobox 1 (PDX-1) to regulate the pancreatic progenitor cell proliferation and maintain mature islet β cell functions. Bisphenol A (BPA) can up-regulate miR-338 to directly inhibit PDX-1 expression, inhibit ATP production, and impair pancreatic islet functions (Wei et al., 2017). In addition, impaired ATP production is also involved in the DNA damage response (DDR) signaling in β cells. Oleson et al. (2019) reported that nitric oxide suppresses DDR signaling in the pancreatic β cell line INS 832/13 cells and rat islets by increasing ATP generation to ameliorate DDR-dependent cell apoptosis. Overall, it is widely accepted that reduced mitochondrial ATP production plays crucial roles in the development of pancreatic β cell dysfunction.

#### 3.1.2 Targeting ATP Synthesis to Improve Pancreatic β Cell Dysfunctions

Thylakoid fragments and lipid molecules had been combined to synthesize a synthetic and biological mixed protein liposome called a highly efficient life-support intracellular opto-driven system (HELIOS). Under the stimulation of red light, HELIOS can increase intracellular ATP concentrations in a variety of cell lines and promote insulin secretion in islet β cells (Zheng et al., 2018). In islets isolated from healthy subjects and diabetic patients, an increase of voltage-dependent anion channel-1 (VDAC1) in the extracellular membrane of mitochondria inhibits ATP synthesis. Treatment with VDAC1 inhibitors can increase ATP production and improve GSIS in isolated islets of type 2 diabetic patients. Administration of VDAC1 inhibitor improved insulin secretion, glucose intolerance and hyperglycemia in obese diabetic mice (Zhang et al., 2019). Studies have also showed that polysaccharides from Portulaca oleracea L (POP) can increase ATP synthesis, change electric potential of cell membrane and mitochondria membrane in INS-1 cells in a voltage-gated Na^+^ channel (VGSC)-dependent manner. Polysaccharides increases intracellular Ca^2+^ level, promotes insulin synthesis and secretion, and increases cellular activity (Hu et al., 2019). In addition, Loureirin B, a flavonoid extracted from Dracaena cochinchinensis, increases insulin secretion and lowers blood glucose levels of diabetic mice. This study further revealed that the promotion of insulin secretion by Loureirin B is mainly achieved through the induction of PDX-1 and V-maf musculoaponeurotic fibrosarcoma oncogene homolog A (MafA) expressions, thus increasing intracellular ATP levels and Ca^2+^ influx (Sha et al., 2018).

Beyond the role in controlling insulin secretion, increased intracellular ATP also regulates the survival and proliferation of islet β cells. Shahrestanaki et al. (2018) found that adenosine can protect Min6 cells against tunicamycin-induced endoplasmic reticulum (ER) stress and enhance insulin secretion. The mechanism is mainly proposed as that adenosine can promote mitochondrial ATP synthesis and increase free Ca^2+^ level in Min6 cells, thereby helping to maintain ER homeostasis, and promoting insulin synthesis and release. Kumar et al. (2018) demonstrated that glucose-stimulated proliferation of pancreatic islet β cells requires the participation of carbohydrate response element-binding protein alpha (ChREBPα), and overexpression of ChREBPα can augment this effect. Further study found that ChREBPα can upregulate the expression and activity of erythroid-derived nuclear factor 2-like 2 (Nrf2), which exerts antioxidant effects, and increases mitochondrial biosynthesis and ATP synthesis (Kumar et al., 2018). Overexpression of Nrf2 *in vitro* promotes the proliferation of islet β cells. Rowley et al. (2017) found that cocoa catechin monomer can promote the nuclear translocation of Nrf2 to stimulate mitochondrial ATP synthesis and improve islet β cell functions. Riopel et al. (2018) reported that flamokines (FKN) can increase ATP content, ATP/ADP ratio and oxygen consumption rate in islet β cells under both basal and glucose stimulation states. Long-term administration of FKN can improve islet β cell dysfunctions and glucose intolerance in diabetic mice. Atanes et al. (2018) found that complement component 3 and 5 (C3 and C5) and the active complement component 3 and 5 receptors (C3aR and C5aR1) are constitutively expressed in the islets of both humans and mice. Activation of C3aR and C5aR1 can increase ATP production and free Ca^2+^ level in islet cells, enhancing GSIS and protecting islets against apoptosis induced by pro-inflammatory factors or palmitic acid. Overall, increased intracellular ATP improves pancreatic β cell functions in multiple pathway.

### 3.2 Extracellular ATP is an Important Regulator of Pancreatic β Cell Functions

In islet β cells, extracellular ATP can promote extracellular Ca^2+^ influx through the ion-channel-linked P2X receptors. Moreover, extracellular ATP also acts on the G protein-coupled P2Y receptors to stimulate Ca^2+^ release from internal calcium storages. The activation of P2Y receptors by extracellular ATP increases the level of inositol troposphere (IP3) in the cytoplasm by activating phospholipase C (PLC), and then IP3 acts on the endoplasmic reticulum IP3 receptor (IP3R) to stimulate Ca^2+^ release from the internal calcium pools. Overall, extracellular ATP activates both P2X and P2Y receptor subtypes to increase the cytosolic free Ca^2+^ levels and promote insulin secretion in pancreatic β cells (Novak, 2008; Dingreville et al., 2019). In rat islets, ATP and zinc could be co-secreted with insulin in response to glucose stimulation, and the GSIS could be inhibited by P2 receptor antagonist (Richards-Williams et al., 2008). In human islets, ATP could be secreted in response to glucose stimulation, and released ATP in return augments GSIS via the activation of P2X receptors. It has been further shown that human islets predominantly express P2X3, P2X5, and P2X7 subtypes of P2 receptors. Released ATP mainly activates P2X3 subtype to increase Ca^2+^ influx and enhance insulin secretion in human islets (Jacques-Silva et al., 2010). During glucose stimulation, extracellular ATP co-secreted with insulin in a pulsatile manner and co-operated with Ca^2+^ and other signals to provide a negative feedback signal driving β-cell oscillations (Bauer et al., 2019).

FAM3A is the first member of family with the sequence similarity 3 (FAM3) (Zhu et al., 2002). Previous studies had demonstrated that FAM3A is a novel mitochondrial protein, and it enhances ATP production in various cell types (Zhang X. et al., 2018; Xu et al., 2019; Chen et al., 2020; Xiang et al., 2020). In diabetic mouse islet β cells, FAM3A expression is significantly reduced. Mice with specific deletion of FAM3A in pancreatic β cells exhibit markedly impaired insulin secretion and glucose intolerance. *In vitro*, FAM3A overexpression enhances whereas FAM3A deficiency blunts ATP production and secretion, and GSIS in primary islets and β cell lines (Yang et al., 2020; Yan et al., 2021). Mechanistically, FAM3A-promoted ATP release activates both P2X and P2Y receptors to increase cellular Ca^2+^, which stimulates the translocation of calmodulin (CaM) from cytoplasm into nucleus and directly interacts with forkhead box protein A2 (FOXA2) to augment its activation effect on the PDX-1 gene expression (Yang et al., 2020). Upregulation of PDX-1 finally induces insulin gene expression and insulin secretion (Yang et al., 2020). Beyond extracellular ATP, intracellular ATP also contributed to FAM3A-induced PDX-1 upregulation and pancreatic β cell proliferation (Yan et al., 2021). Moreover, Weitz et al. (2018) also reported that ATP is a potent activator for islet macrophages, and mouse islet macrophages utilize locally released ATP to regulate islet β cell activity.

Taken together, both intracellular and extracellular ATP play important roles in regulating insulin synthesis and secretion, cell survival and proliferation of pancreatic β cells and hepatocyte. Extracellular ATP plays important roles in regulating the expression of PDX-1 gene, which is the master regulator of pancreatic β cell functions. Impaired ATP production and release are central events of pancreatic β cell dysfunctions. To restore ATP production and release holds great promise for correcting pancreatic β cell dysfunctions and treating diabetes.

## 4 ATP Regulates Hepatic Glycolipid Metabolism

### 4.1 Reduced Hepatic ATP Synthesis is Highly Associated With Hepatic Glycolipid Metabolism

In the liver, abnormal ATP synthesis is highly associated with dysregulated glycolipid homeostasis (Gonzales et al., 2010). By using ^31^P magnetic resonance spectroscopy (MRS), it had been revealed that the hepatic ATP content in type 2 diabetic patients is reduced compared with health subjects (Szendroedi et al., 2009). Additional, reduced hepatic ATP content is tightly correlated with insulin resistance, obesity and fasting hyperglycemia (Nair et al., 2003; Szendroedi et al., 2009; Schmid et al., 2011). Furthermore, hepatic ATP content is also significantly decreased in type 1 diabetic mice and type 2 obese diabetic mice (Miyamoto et al., 2008; Berglund et al., 2009; Wang et al., 2014a; Wu et al., 2019). Amelioration of hyperglycemia and steatosis by factors such as exercise, melatonin and eicosapentaenoic acid (EPA) treatment are always associated with increased hepatic ATP content in obese diabetic mice (Wang et al., 2014a; Stacchiotti et al., 2016; Echeverria et al., 2019). It is well known that excessive intake of fructose and sugar contributes to the rising prevalence of non-alcoholic fatty liver disease (NAFLD) parallels with the rise in obesity and diabetes (Mai and Yan, 2019). Researchers revealed that fructose metabolism via fructokinase C gives rise to ATP over-consumption, which led to nucleotide turnover and excessive uric acid generation (Mai and Yan, 2019). Other studies also elucidated carbohydrate response element-binding protein (ChREBP) can protect against the ATP over-consumption induced by fructose through L-type pyruvate kinase (Shi et al., 2020). Hepatic ATP recovery from depletion induced by fructose injection is inversely correlated with body mass index (BMI) (Cortez-Pinto et al., 1999). Meanwhile, when compared with healthy control, the obesity-related NASH patients were harder recovery from the hepatic ATP depletion (Cortez-Pinto et al., 1999). Consequently, hepatic ATP depletion causes oxidative stress and mitochondrial dysfunction, and inhibition of protein synthesis (Jensen et al., 2018; Mai and Yan, 2019). These changes finally trigger obesity, insulin resistance, hepatic lipid deposition, and metabolic syndrome (Jensen et al., 2018; Mai and Yan, 2019). A recent study showed that chronic HFD feeding aggravates the metabolic disorders by inhibiting ATP production in mice, and the reduction of ATP synthesis decreases ATP/ADP ratio, causes mitochondrial dysfunction, and exacerbates lactate dehydrogenase (LDH) levels, which further leads to altered expressions of glucose transporters (GLUTs) in multiple metabolic tissues (Jha and Mitra Mazumder, 2019).

At present, there is increasing evidence that ATP deficiency is involved in the development of liver insulin resistance, glucose and lipid metabolism disorders, and even diabetes. Clearly, to restore hepatic ATP synthesis might be a potential therapeutic strategy for the treatment of metabolic disorders. It has been reported that liver-targeted Nano-MitoPBN, which can not only decrease hepatic glucose output but also maintain normal mitochondrial bioenergetics function and ATP production, could be a promising drug for treating diabetes (Wu et al., 2019). There are three paralogous ANT genes, Ant1, Ant2 and Ant4 in mice. It has been reported that targeted knockout of Ant2 in mouse liver or systemic administration of low-dose carboxyatractyloside, a specific inhibitor of AAC, enhances uncoupled respiration without affecting mitochondrial integrity, ATP synthesis, and liver functions, thus improving hepatic steatosis, obesity and insulin resistance in diabetic mice (Cho et al., 2017). So far, the concise and direct mechanisms of intracellular ATP in regulating hepatic glycolipid metabolism still remains largely unclear.

### 4.2 Intracellular ATP on Glucose and Lipid Metabolism in Hepatocytes

Intracellular ATP in hepatocytes ensures the energy supply for various life activities and biological functions, and it is the direct source of energy in cells (Mookerjee et al., 2017). It had been reported that staurosporin and CD95 stimulation caused hepatocyte necrosis when intracellular ATP levels were reduced (Leist et al., 1997). It had also been reported that increased apoptosis of hepatocyte promoted liver fibrosis, while targeted apoptosis of stellate cells reduced or even reversed liver fibrosis (Fallowfield and Iredale, 2004; Kim et al., 2005b; Oakley et al., 2005). Moreover, mitochondrial dysfunction plays pivotal roles in the development of hepatic glucose and lipid deregulation (Fujita et al., 2008; Koliaki et al., 2015; Mansouri et al., 2018; Lee et al., 2019). Impaired mitochondrial oxidative phosphorylation reduced intracellular ATP content, which led to cellular dysfunction, decreased β-oxidation levels of free fatty acids, increased lipogenesis and produced large amounts of reactive oxygen species (ROS) (Koliaki et al., 2015; Mansouri et al., 2018; Lee et al., 2019). Overall, although reduced intracellular ATP content is highly associated with metabolic diseases, its underlying mechanism(s) remains largely unclear.

### 4.3 Extracellular ATP is an Important Regulator of Hepatic Glycolipid Metabolism

Purinergic receptors also been detected in the hepatocytes, and regulate hepatic metabolic processes (Jain and Jacobson, 2021). ATP administration ameliorated hyperglycemia and alleviated fatty liver in diabetic rats (Tahani et al., 1977). Starved rat hepatocytes treated with high dose of extracellular ATP elevated the intracellular ATP adenosine levels to suppress gluconeogenesis (Asensi et al., 1991). In hepatocytes and liver, extracellular ATP also promote glycogenolysis (Keppens and De Wulf, 1985, 1986; Haussinger et al., 1987), and glycogen phosphorylase activated by purinergic agonists as a dose-dependent manner in isolated rat hepatocytes (Keppens and De Wulf, 1985). Other study has shown that P2Y1 agonist 2-methylthioadenosine 5′-diphosphate (2-MeSADP) could stimulate glycogen phosphorylase and elevate cytosolic free Ca^2+^ levels in primary rat hepatocytes (Dixon et al., 2004). In primary human hepatocytes, extracellular ATP increases the cytosolic free Ca^2+^ level and stimulated glycogen phosphorylase in a dose-dependent manner via P2Y2 receptor (Dixon et al., 2005). The P2X4/P2X7 receptor agonist 2′3′-O-(4-benzoyl-benzoyl)-adenosine 5′-triphosphate (BzATP) reduced the hepatic glycogen content indicated the P2X receptor also involved in glycogen metabolism (Emmett et al., 2008).

Primary rat hepatocytes treated with extracellular ATP exhibited decreased activity of acetyl-CoA carboxylase (ACC) and repressed *de novo* fatty acid synthesis, and this process might be mediated by Ca^2+^ (Guzman et al., 1996). Meanwhile, the activity of carnitine O-palmitoyltransferase I (CPT-1) is also be inhibited by extracellular ATP through a PKC-dependent pathway in the same study (Guzman et al., 1996). Aa a non-thienopyridine ATP analog, acute intravenous cangrelor injection increased HDL uptake and biliary bile acid secretion in mouse liver, and these effects were blunted in P2Y13 deficient mice (Fabre et al., 2010). Additional study using longer cangrelor treatment also exhibited the same results, suggesting that P2Y13 might be a potential therapeutic target for HDL metabolism (Serhan et al., 2013).

### 4.4 Hepatocyte-Released ATP Suppresses Gluconeogenesis and Lipogenesis Independent of Insulin

As discussed above, hepatic ATP content is significantly decreased in animals and humans under diabetic conditions, suggesting that restoration of hepatic ATP content might be a potential strategy for treating diabetes and fatty liver.

As ATPSβ is the key catalytic subunit of ATPS, we found that in STZ-induced type 1 diabetic, and obese type 2 diabetic mouse livers, the expression of ATPSβ is decreased with reduced ATP content (Wang et al., 2014a). Hepatic overexpression of ATPSβ markedly inhibits gluconeogenesis, improves insulin resistance and hyperglycemia, and attenuates steatosis with increased hepatic ATP content in obese diabetic mice (Wang et al., 2014a). Mechanically, ATPSβ overexpression increases ATP production and release in hepatocytes, the released ATP activates P2X receptors to induce the influx of extracellular Ca^2+^, and P2Y receptors to trigger the Ca^2+^ release from internal calcium storages, increasing cellular Ca^2+^ level to activate CaM. Upon activation, CaM directly activates PI3K-Akt pathway independent of insulin to promote the nuclear exclusion of forkhead box protein O1 (FOXO1), the key transcription factor that controls the expression of gluconeogenic genes, and repress gluconeogenic gene expression and gluconeogenesis in hepatocytes (Wang et al., 2014a).

FAM3A as a mitochondrial protein increased intracellular and extracellular ATP production to activates P2 receptors-Ca^2+^-CaM pathway, then promote protein kinase B (Akt) to represses the expression of gluconeogenic and lipogenic genes in obese diabetic mouse livers. This process is independent of insulin (Wang et al., 2014b). Further study shows that hepatic FAM3A is directly repressed by miR-423-5p under diabetic condition (Yang et al., 2017). Hepatic miR-423-5p overexpression inhibits FAM3A-ATP-P2R signaling pathway to promote hyperglycemia, insulin resistance and steatosis in normal mice, while miR-423-5p inhibition activated FAM3A-ATP-P2R signaling pathway to ameliorate hyperglycemia and fatty liver in obese mice (Yang et al., 2017). In the livers of NAFLD patients as well as obese diabetic mice, the expressions of nuclear factor erythroid-derived 2 (NFE2) and miR-423-5p are increased with decreased FAM3A expression, suggesting that inhibition of FAM3A-ATP-P2R pathway by NFE2/miR-423-5p axis is involved in the development of NAFLD and diabetes in both humans and animals (Yang et al., 2017). Moreover, it has been reported that activation of FAM3A-ATP-P2R pathway due to the inhibition of NFE2/miR-423-5p axis in the liver is involved in exercise-induced improvement of insulin resistance and hyperglycemia in diabetic animals (Zhang Y. et al., 2018). In patients with obesity, reduction of body weight after bariatric surgery is associated with reduced circulating miR-423-5p level (Bae et al., 2019). In type 2 diabetic patients, amelioration of hyperglycemia after aerobic and resistance training is also associated with decrease circulating miR-423-5p level (Olioso et al., 2019). Disheveled binding antagonist of beta catenin 1 (Dapper1), the key factor in Wnt signaling, can elevate stimulate ATP production and release in diabetic mouse liver and cultured hepatocytes. Dapper1-induced ATP releases similarly activates p110α/Akt pathway via the P2 receptors to repress gluconeogenesis and lipogenesis in hepatocytes (Kuang et al., 2017). These findings have established that hepatocyte-released ATP is an important regulator of glucose and lipid metabolism *via* the activation of P2 receptors.

Beyond regulating glucose and lipid metabolism, FAM3A-ATP-P2R pathway also exerts beneficial effects on ischemia/reperfusion injury (IRI) in mouse livers (Chen et al., 2017) and inhibition of FAM3A-ATP-P2R pathway is involved in the development of adipocyte dysfunction (Chi et al., 2017), which also plays important roles in the pathogenesis of diabetes. Furthermore, we found that antidepressive drug doxepin can activate FAM3A-ATP signaling pathway to ameliorate hyperglycemia and fatty liver in HFD mice and db/db mice (Chen et al., 2020). Notably, doxepin’s beneficial effects on hyperglycemia, fatty liver and obesity were completely abolished in FAM3A-deficient mice. Given that depression is the risk of diabetes and fatty liver (Richardson et al., 2008; Youssef et al., 2013), doxepin may be prescribed in priority to depressive patients with diabetes or fatty liver as anti-depressant. Meanwhile, dulaglutide, an agonist of glucagon-like peptide-1 receptor (GLP-1A), promoted lipolysis and fatty acid oxidation by activating FAM3A to attenuate steatosis (Lee et al., 2022). So far, the mechanism of FAM3A-induced ATP synthesis and release still remains unrevealed. To solve these issues will definitely shed light on the pathogenesis and treatment of mitochondrial dysfunctions, diabetes and steatosis.

Overall, both intracellular and extracellular ATP play important roles in regulating hepatic glucose and lipid metabolism. Particularly, hepatocyte-released ATP is an important signaling molecule that regulates the activities of CaM, Akt and FOXO1, the expression of gluconeogenic gene expression and gluconeogenesis in hepatocytes, particularly in basal condition when serum insulin level is low. Impaired ATP production and release contribute much to the development of oxidative stress, insulin resistance, hyperglycemia and steatosis.

## 5 Summary and Perspective

ATP plays decisive roles in regulating the metabolic functions of pancreatic β cell and liver *via* the intracellular and extracellular signaling transduction. Impaired mitochondrial ATP production induced by free fatty acids, glucose and pro-inflammatory cytokines is one of the key events in the pathogenesis of pancreatic β cell dysfunctions and dysregulated hepatic glycolipid metabolism.

**FIGURE 2:**
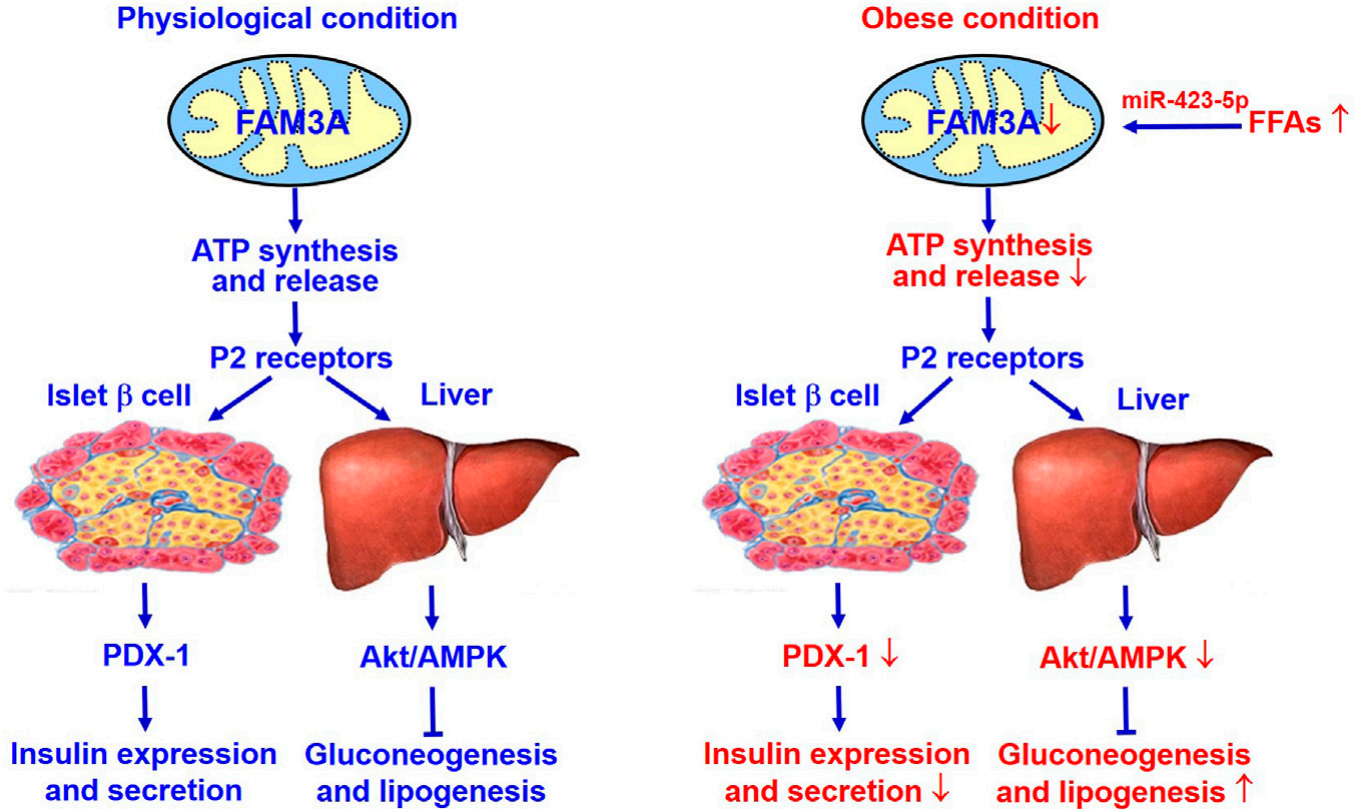
FAM3A is a novel target for treating diabetes and NAFLD. FAM3A is a new mitochondrial protein that enhances ATP production and release in pancreatic β cells and hepatocytes. In physiological condition, FAM3A-ATP-P2R signaling pathway plays important roles in controlling PDX-1 and insulin expressions in pancreatic β cells, and suppressing gluconeogenesis and lipogenesis independent of insulin in liver. Under diabetic condition, inhibition of FAM3A-ATP-P2R signaling pathway causes pancreatic β dysfunctions, and increases hepatic gluconeogenesis and lipogenesis. Clearly, activating FAM3A-ATP-P2R signaling pathway represents a novel strategy for treating T2DM and NAFLD. Akt, protein kinase B; ATP, adenosine triphosphate; FFAs, free fatty acids; NAFLD, non-alcoholic fatty liver disease. P2R, purinergic P2 receptors (ATP, ADP and UTP as ligands); PDX-1, pancreatic and duodenal homeobox 1.

The new mitochondrial protein FAM3A suppresses hepatic gluconeogenesis and lipogenesis independent of insulin, and also controls PDX1 and insulin expressions in pancreatic β cells. Under diabetic condition, activation of hepatic FAM3A expression has benefit for ameliorating hyperglycemia. Activation of FAM3A in pancreatic β cells up-regulates PDX-1 and insulin expression, and enhances insulin secretion. Clearly, activating FAM3A-ATP-P2R pathway represents a novel strategy for treating diabetes and other metabolic diseases (Figure 2).
